# The hyaluronan synthesis inhibitor 4-methylumbelliferone exhibits antitumor effects against mesenchymal-like canine mammary tumor cells

**DOI:** 10.3892/ol.2013.1124

**Published:** 2013-01-10

**Authors:** TERUYOSHI SAITO, TAMURA DAI, RYUJI ASANO

**Affiliations:** Laboratory of Veterinary Pharmacology, Nihon University College of Bioresource Science, Fujisawa, Kanagawa 252-0880, Japan

**Keywords:** 4-methylumbelliferone, hyaluronan, hyaluronan synthase, apoptosis, chemokinesis, chemotaxis, canine mammary tumor

## Abstract

Hyaluronan (HA), a principal constituent of the extracellular matrix (ECM), mediates growth and metastasis of tumor cells. The role of HA in the epithelial-mesenchymal transition (EMT) is well known, and increased ECM remodeling is observed in mesenchymal-like cells. The HA synthesis inhibitor 4-methylumbelliferone (4-MU) is anti-tumorigenic for various malignant tumors. However, the antitumor effect of 4-MU against canine mammary tumor cells that possess a mesenchymal-like phenotype is unclear. We examined the antitumor effect of 4-MU on CF41.Mg mesenchymal-like canine mammary tumor cells. We investigated the influence of 4-MU on the expression of *HA synthase (HAS) 1-3* mRNA and observed dose-dependent downregulation of *HAS2* mRNA at 24-72 h; in contrast, *HAS3* expression was elevated at 24 h. Thus, 4-MU inhibited HA synthesis via HAS2 repression. 4-MU also inhibited cell proliferation and induced apoptosis in the CF41.Mg cells. Our experiments showed that 4-MU-induced apoptosis in CF41.Mg cells involved induction of the pro-apoptotic gene *BAX*. We also assessed motility and found that 4-MU reduced chemokinesis and chemotaxis in CF41.Mg cells. Our data suggest that 4-MU may serve as a candidate therapeutic agent for the treatment of canine mammary tumors. Since 4-MU exhibits antitumor activity in mesenchymal-like cells, it may also be a useful inhibitor of canine mammary tumor invasion and metastasis.

## Introduction

Hyaluronan (HA) is a non-sulfated linear glycosaminoglycan present in the extracellular matrix (ECM) of most tissues. It is synthesized and extruded at the plasma membrane by HA synthases (*HAS1*, *HAS2* and *HAS3*) and consists of repeating *d*-glucuronic acid and *N*-acetyl-*d*-glucosamine units (1,2). Several studies have shown that HA plays important roles in matrix assembly, cell proliferation, differentiation and migration during development and disease (3). Previous studies have shown that elevated HA in the tumor stroma correlates with tumor aggressiveness and poor prognosis in patients with breast, prostate and ovarian cancers (2,4). Knockdown of HAS genes in cancer cells inhibits proliferation, invasion, and motility *in vitro* and tumor growth and metastasis *in vivo*(5). In breast cancer cells, *HAS2* expression is often strongly correlated with malignant behavior (6–8). Thus, abnormalities of HA synthesis and/or degradation are frequently observed in various cancers.

4-methylumbelliferone (4-MU; 7-hydroxy-4-methyl-2H-1-benzopyran-2-one) was first found to specifically inhibit HA synthesis in human skin fibroblasts (9). It does so by causing substrate inhibition of *HAS*s due to 4-MU binding to GlcUA via UDP-GlcUA (10). 4-MU also inhibits HA synthesis via repression of *HAS2* and/or *HAS3* mRNA in breast cancer, melanoma and ovarian cancer cells (11). 4-MU-mediated inhibition of HA synthesis produces anticancer effects on cell proliferation, migration, invasion and metastasis *in vitro* and *in vivo* in several human cancers such as breast and prostate cancers (11–15). As it produces the anticancer effect without causing severe side effects, 4-MU has the potential to become a novel anticancer drug. However, it remains unclear whether 4-MU exhibits anticancer activity against canine mammary tumor cells.

Metastasis is the primary cause of mortality in various human and canine cancers. Metastatic cells exhibit elevated cell motility, which mediates the epithelial to mesenchymal transition (EMT). In general, cell motility may be categorized as chemokinesis and chemotaxis. Chemokinesis is random cell movement, which involves the separation of tumor cells from their primary site and is thus important during the EMT process (16,17). Chemotaxis is defined as a directional cell movement. Once ECM remodeling has been activated, mesenchymal-like cancer cells have many opportunities for interaction with components of the ECM such as HA, collagen and laminin (18–20).

Canine mammary tumors are one of the most frequent cutaneous tumors of female dogs. Histologically, approximately 50% of canine mammary tumors are malignant, and metastases and/or recurrences are common causes of mortality in these animals (21,22). Recent studies of human and canine gene expression in tumor and normal mammary samples suggest many cancer-related genes that are deregulated in human breast cancer are also found in canine mammary tumors (23). For example, in malignant mammary tumors in dogs, the expression patterns of ECM remodeling-related genes are very similar to those in humans (23). Canine mammary tumors are classified based on cytological characteristics as epithelial, mesenchymal or mixed, according to origin. Histologically complex carcinoma is commonly observed in canine mammary tumors. In benign canine mammary tumors, complex adenomas and benign mixed tumors are most common. This histological type has both epithelial and mesenchymal (myoepithelial) components (24). However, it is not clear whether 4-MU acts as an antitumor agent against mesenchymal cells in canine mammary tumors. The aim of this study was, therefore, to define the antitumor effect of 4-MU on CF41.Mg cells with properties of mesenchymal-like canine mammary tumor cells.

## Materials and methods

### 4-MU

4-MU was purchased from Wako Pure Chemicals (Osaka, Japan). The 4-MU stock solution was dissolved in DMSO. The final concentration of DMSO in the medium was adjusted to 0.1% in all experiments.

### Cell culture

Canine mammary tumor cell line CF41.Mg and CF33 cells were obtained from the American Type Culture Collection (Manassas, VA, USA). Both were cultured in Dulbecco’s modified Eagle’s medium (DMEM) (Nissui, Tokyo, Japan) supplemented with 10% heat-inactivated fetal bovine serum (FBS), 4 mM l-glutamine, 10 mg/ml streptomycin and 10,000 U/ml penicillin G. The cells were maintained at 37°C in a humidified atmosphere with 5% CO_2_.

### Cell proliferation analysis

We used the Cell Counting Kit-8 (Dojindo Laboratories, Kumamoto, Japan) to assess the effect of 4-MU on cell proliferation. CF41.Mg cells were plated in 96-well plates (4.5×10^3^ cells/well). At each time point (days 0–4), 10 *μ*l CCK-8 reagent was added, and the plates were incubated for 4 h. After incubation, the absorbances were measured at 450 nm with a Benchmark plus microplate reader (Bio-Rad, Tokyo, Japan). In these experiments, 5 replicate wells were used for each time point; the results are presented as means ± SD.

### Cell cycle and apoptosis analysis

Cells were harvested and washed with phosphate-buffered saline (PBS), resuspended in 70% ethanol in distilled water, and kept at −30°C overnight. Before analysis, cells were mixed and incubated for 30 min in PBS containing 0.05 mg/ml propidium iodide (PI) and 100 U/ml RNase A. The suspension was filtered through a 5-ml polystyrene round-bottom tube with a cell-strainer cap (Becton Dickinson, Franklin Lakes, NJ, USA) and analyzed by FACSCalibur (Becton Dickinson) and Flow-Jo 7 software (Tree Star, Ashland, OR, USA).

### Real-time RT-PCR

Total RNA was extracted from cells using the TRIzol reagent (Invitrogen, Carlsbad, CA, USA), and cDNAs were synthesized with a PrimeScript™ RT Master Mix (Takara Bio, Shiga, Japan) according to the manufacturer’s protocols. Real-time PCR was performed with SYBR Premix Ex *Taq*™ (Takara Bio) and the ABI Prism 7500 Real-Time PCR System (Applied Biosystems, Foster City, CA, USA) under the following conditions: 95°C for 30 sec; 40 cycles of 95 kC for 5 sec and 60°C for 34 sec. Specific primer sets for *BAX* (forward, 5′-CGCATCGGAGATGAACTGGA-3′; reverse, 5′-ACCAGTTTGCTGGCAAAGTAGAAG-3′) and *N-cadherin* (forward, 5′-AGGAATCCGACGATTGGA TGAG-3′; reverse, 5′-GTGGGATCATTGTCAGCAGCT TTA-3′) were purchased from Takara Bio. *HAS1* (forward, 5′-GGACTACGTGCAGGTGTGTG-3′; reverse, 5′-CTCAC CTAGGGGACCACTGA-3′), *HAS2* (forward, 5′-CTTAGA GCACTGGGA-3′; reverse, 5′-TCTAAAACT TTCACCA-3′), *HAS3* (forward, 5′-AAGTAGGGGGAG TTGG-3′; reverse, 5′-CCCAGAGGCCCACTAA-3′), *vimentin* (forward, 5′-ATTGCTCTGCCTCTTC-3′; reverse, 5′-GGCAAG CTT CACTCAA-3′), *E-cadherin* (forward, 5′-CCCTCATTATAG CCAT-3′; reverse, 5′-AGTCCATATTTCGAGG-3′), and *GAPDH* (forward, 5′-AAGGCTGAGAACGGGA-3′; reverse, 5′-GGAGGCATTGCTGACA-3′) were obtained from Operon Biotechnology (Tokyo, Japan). The specificity of each amplification was confirmed by a dissociation curve consisting of a single peak. All samples were amplified in triplicate in each experiment. The values were normalized to GAPDH. Relative levels of mRNA were calculated using the ΔΔCt method.

### Motility assay

To investigate the effect of 4-MU on chemokinesis and chemotaxis, the Boyden chamber migration assay was employed (25,26). Before the motility assay, cells were starved overnight in DMEM supplemented with 1% FBS. CF41.Mg cells (1.5×10^4^ cells/well) treated with 4-MU for 24 h were loaded in the upper chambers of polycarbonate membrane transwell inserts (Corning Inc., Corning, NY, USA). The Boyden chamber contained two medium-filled compartments. Each chamber (upper/lower) contained a different concentration (1%/1%, and 1%/10%) of FBS. Each set of lower and upper chambers was separated by an 8-*μ*m pore size polycarbonate membrane. The cells were allowed to migrate for 10 h. The membranes were then fixed with 4% paraformaldehyde phosphate buffer solution (Wako) and stained with Meyer’s hematoxylin (Wako). The cells on the upper side of each membrane were removed with cotton swabs. The cells on the lower side were counted under a light microscope at ×200 magnification. Four random microscopic fields were counted. Results are presented as means ± SD.

### Statistical analysis

The statistical significance of differences in chemokinesis and chemotaxis were determined by Student’s t-test. P<0.05 was considered to indicate a statistically significant result.

## Results

### Canine mammary tumor CF41.Mg cells have properties of mesenchymal-like cells

**Figure 1:**
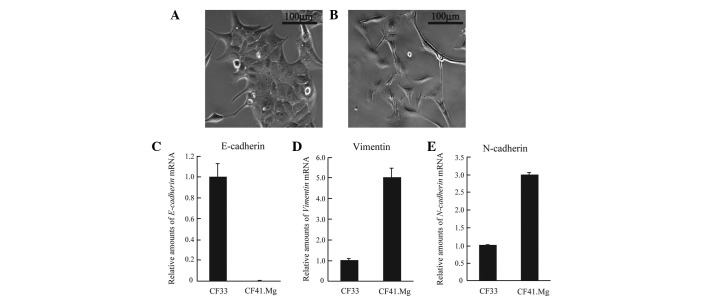
CF41.Mg cells show a mesenchymal-like phenotype. (A) and (B) Representative images of CF33 and CF41.Mg cells, respectively. Magnification, ×100. (C–E) Transcript levels of E-cadherin, vimentin and N-cadherin were analyzed by real-time RT-PCR in CF33 and CF41.Mg cells. Data represent means ± SD of triplicate experiments.

During tumor progression, advanced tumor cells frequently exhibit a conspicuous loss of cell-cell adhesion such as downregulation of E-cadherin. The loss of epithelial features is accompanied by increased motility, resistance to anti-cancer drugs, and expression of mesenchymal genes such as vimentin and N-cadherin (16,27). These processes are known as EMT, and are thought to be critical to cancer cell invasion and metastasis. To examine the effect of 4-MU on mesenchymal-like cells of canine mammary tumors, we first determined whether canine mammary tumor CF41.Mg cells possess features characteristic of epithelial or mesenchymal cells. First, cell morphology was examined by microscopy. CF41.Mg displayed highly elongated mesenchymal morphology, whereas canine mammary tumor CF33 cells showed epithelial morphology and formed cell-cell attachments (Fig. 1A and B). Next, molecular markers of cell origin such as E-cadherin, vimentin and N-cadherin were investigated. CF41.Mg cells expressed markedly lower levels of E-cadherin (an epithelial marker) than did CF33 (Fig. 1C). Furthermore, CF41.Mg exhibited higher levels of vimentin and N-cadherin (mesenchymal markers; Fig. 1D and E). Thus, CF41.Mg cells have a mesenchymal-like phenotype in canine mammary tumor cell lines. To evaluate the antitumor activity of 4-MU (via cell proliferation, apoptosis and motility), we used CF41.Mg canine mammary tumor cells as a model of the morphology and characteristics of mesenchymal-like cells.

### 4-MU inhibits HA synthesis by downregulating HAS2 mRNA expression

**Figure 2:**
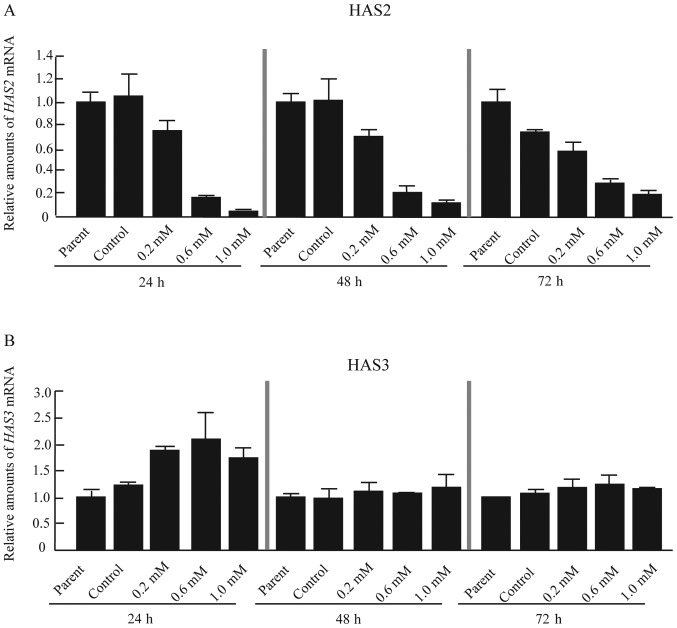
4-methylumbelliferone (4-MU) altered expression of *HAS2* and *HAS3* mRNA in CF41.Mg cells. Transcript levels of *HAS2* and *HAS3* were analyzed by real-time RT-PCR in CF41.Mg cells treated with 0.2, 0.6 and 1.0 mM 4-MU. The data represent means ± SD of triplicate experiments. Real-time RT-PCR of (A) *HAS2* and (B) *HAS3* mRNA expression in CF41.Mg cells treated with the vehicle (Control) or different concentrations of 4-MU (0.2, 0.6 1.0 mM) at 24–72 h. Untreated cells are marked ‘Parent.

In mammalian cells, HA is produced at the plasma membrane by three *HAS*s (*HAS1-3*). Recently, Kultti *et al* reported that 4-MU inhibits HA synthesis by transcriptional repression of *HAS2*, *HAS3* or both in human breast cancer cell lines (11). To determine the effect of 4-MU on HA synthesis in CF41.Mg cells, the expression of *HAS1-3* mRNA was analyzed. *HAS1* mRNA was undetectable by real-time RT-PCR (data not shown). The data therefore indicated that CF41.Mg cells principally synthesized HA by *HAS2* and *HAS3* (Fig. 2A and B). CF41.Mg cells treated with 4-MU showed a dose-dependent reduction in *HAS2* mRNA expression (Fig. 2A). In contrast, *HAS3* mRNA was induced 24 h after treatment with 4-MU (Fig. 2B); this effect disappeared by 48 and 72 h (Fig. 2B). Therefore, 4-MU inhibited HA synthesis through repression of *HAS2* mRNA in CF41.Mg cells.

### 4-MU markedly inhibited growth arrest and apoptosis of CF41.Mg cells

**Figure 3:**
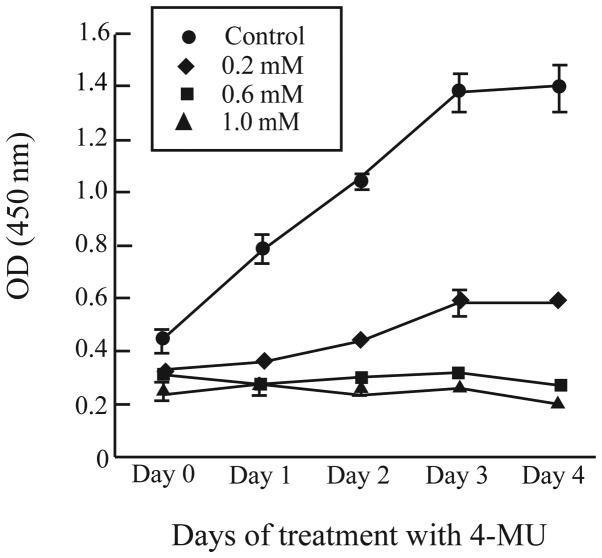
4-methylumbelliferone (4-MU) markedly inhibited cell proliferation in a dose-dependent manner. The effects of 4-MU on cell proliferation were analyzed by WST-8 assays from days 0 to 4 of culture. Data are means ± SD (n=5). Closed circles represent cells treated with vehicle (Control). Cells treated with a different concentrations of 4-MU (0.2, 0.6 and 1.0 mM) are indicated by closed rhombuses, closed squares and closed triangles, respectively.

**Figure 4:**
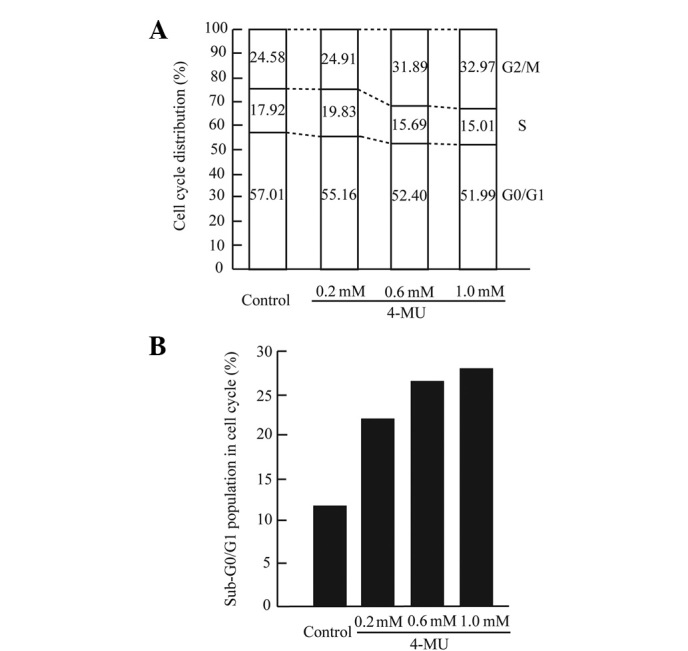
4-methylumbelliferone (4-MU) led to an increase in apoptotic cells and an alteration in the distribution of S-phase and G2/M phase CF41.Mg cells. The effects of 4-MU on cell cycle distribution were estimated by flow cytometry. (A) The percentage of cells distributed in each cell-cycle stage. Cells were treated with vehicle (Control) or 4-MU (0.2, 0.6 or 1.0 mM) for 72 h. (B) Flow cytometry of apoptotic cells treated with the vehicle (Control) or different concentrations of 4-MU (0.2, 0.6 and 1.0 mM) at 72 h. Data from 20,000 cells were analyzed by Flow-Jo 7 in this experiment.

**Figure 5:**
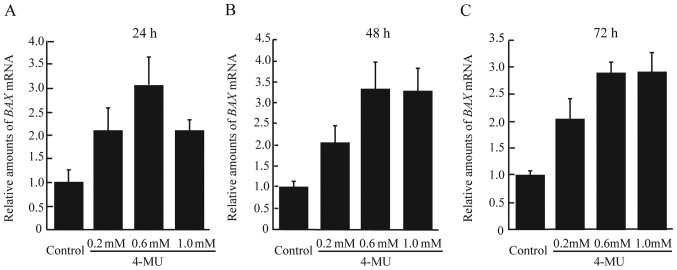
4-Methylumbelliferone (4-MU) increased the expression of proapoptotic gene *BAX*. To confirm the effect of 4-MU on apoptosis-related genes, we measured *BAX* mRNA expression by real-time RT-PCR. (A–C) Levels of BAX mRNA in CF41.Mg cells treated with vehicle (Control) or 4-MU (0.2 mM, 0.6 mM, or 1.0 mM) were measured using real-time RT-PCR at 24-72 h. Data are means ± SD of triplicate experiments.

In human breast cancer cells, the rate of cell proliferation often correlates with HA synthesis and *HAS2* expression (8). Furthermore, 4-MU inhibits cell proliferation in various cancer cells (11,12). To analyze the effect of 4-MU on cell proliferation in CF41.Mg cells, we used a quantitative WST-8 assay upon addition of 4-MU and at 0, 1, 2, 3 and 4 days (Fig. 3). The number of cells in control cultures increased steadily during the days after plating, while proliferation was markedly suppressed by 0.2, 0.6 and 1.0 mM 4-MU (Fig. 3). Proliferation of CF41.Mg cells was completely blocked by 0.6 and 1.0 mM 4-MU (Fig. 3). Recently, Lokeshwar *et al* reported that human prostate cancer PC3-ML cells exhibited a change in cell morphology within 2 days after 4-MU treatment (12). However, CF41.Mg cells showed no changes even 4 days after treatment with 0.2, 0.6 and 1.0 mM 4-MU (data not shown). Thus, 4-MU inhibited growth of CF41.Mg cells, as it does for several human cancer cells. 4-MU markedly inhibited proliferation of CF41.Mg cells in the experiments; to determine the effect of 4-MU on cell cycle distribution and apoptosis, we used flow cytometry in cultures treated with 4-MU for 24, 48 and 72 h. Within 48 h of treatment with each concentration of 4-MU, CF41.Mg showed no marked changes in cell cycle distribution and apoptosis (data not shown). After 72 h exposure to 4-MU (0.6 and 1.0 mM), cell numbers in G2/M phase were slightly increased, and the number of S-phase cells decreased in a dose-dependent manner (Fig. 4A). After G2/M arrest, many cancer cell lines, notably certain breast cancer cell lines, exhibit morphological changes consistent with apoptosis (28). To determine the effect of 4-MU on apoptosis in CF41.Mg cells, the percentage of apoptotic cells in our specimens was quantified with PI staining and flow cytometry, with the sub-G0/G1 peak representing apoptotic cells. Cells treated with 4-MU (0.2, 0.6 and 1.0 mM) showed percentages of apoptotic cells that were approximately 2 times higher than control cells (Fig. 4B). To clarify the effect of 4-MU on apoptosis-related genes, the expression of *BAX* mRNA was measured using real-time RT-PCR. As shown in Fig. 5A–C, 4-MU-treated cells demonstrated higher levels of *BAX* mRNA expression after 24–72 h. Therefore, 4-MU inhibited cell proliferation mainly through the induction of apoptosis. It is possible that the 4-MU-treated cells showed no change in cell cycle distribution at 24 and 48 h due to the lapse in time between mRNA expression and protein synthesis.

### 4-MU reduces chemokinesis and chemotaxis of CF41.Mg cells

**Figure 6:**
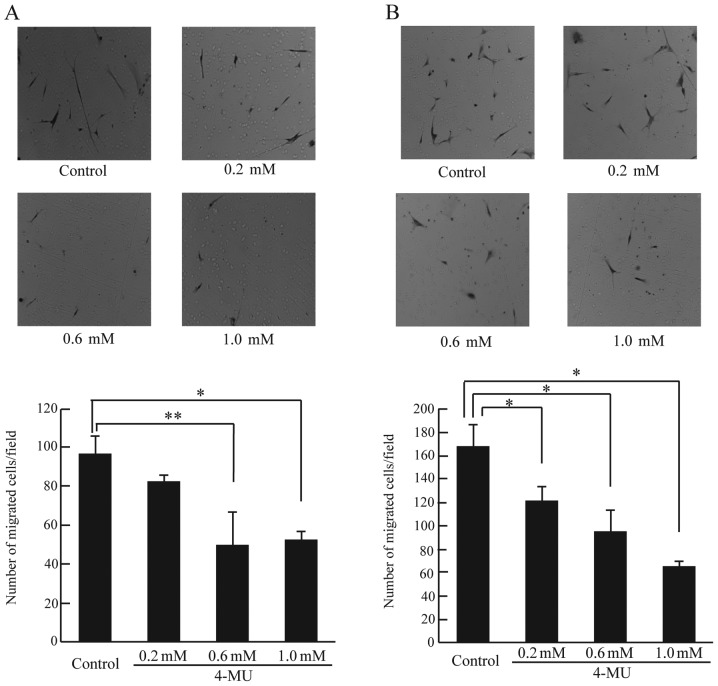
4-methylumbelliferone (4-MU) reduced both chemokinesis and chemotaxis of CF41.Mg cells. (A) The effect of 4-MU on chemokinesis of CF41.Mg cells was assessed by Boyden chamber assays. Representative images of a migration assay for migrated CF41.Mg cells treated with vehicle or 4-MU are shown. Magnification, ×200. Data shown are means ± SD (n=4). ^**^P<0.05, ^*^P<0.01, vs. control, using the Student’s t-test. (B) The effect of 4-MU on chemotaxis of CF41.Mg cells was assessed by Boyden chamber assays. Representative images of a migration assay for migrated CF41.Mg cells treated with vehicle or 4-MU are shown. Magnification, ×200. Data are means ± SD (n=4). ^*^P<0.01 vs. control, using the Student’s t-test.

It is well known that increased cell motility is essential for cancer cell metastasis. Cell motility can be divided into two types, namely random cell motility (chemokinesis) and directional cell motility (chemotaxis). Chemokinesis and chemotaxis play an important role in cancer invasion and metastasis (17,25,29). To investigate the effect of 4-MU on chemokinesis and chemotaxis in CF41.Mg cells, a Boyden chamber assay was used. As shown in Fig. 6A, chemokinesis in cells treated with 0.6 and 1.0 mM 4-MU was significantly reduced compared to control cells (Fig. 6A). Furthermore, cells treated with 4-MU at all concentrations showed markedly reduced chemotaxis (Fig. 6B). 4-MU reduced cell motility (chemokinesis and chemotaxis) in CF41.Mg cells; it is possible that 4-MU could prevent the invasion and metastasis of canine mammary tumor cells.

## Discussion

Previous studies have reported that 4-MU acts as a tumor suppressor against various cancers (11–15). However, it is not clear whether 4-MU shows anticancer effects against mesenchymal-like cells derived from canine mammary tumors. Our results revealed that 4-MU inhibited HA synthesis via reduction of *HAS2* mRNA levels, as well as conspicuous growth inhibition, apoptosis associated with *BAX* mRNA, and reduction of chemokinesis and chemotaxis. Thus, 4-MU is an anticancer agent that inhibits cell growth and cell motility of mesenchymal-like canine mammary tumor cells.

HA is one of the major components of ECM and is essential for embryonic development and wound healing in normal tissue. HA also plays an important role in cancer cell proliferation, angiogenesis, invasion and metastasis. Increased levels of HA in the stroma or serum are associated with malignancy in human patients with breast and ovarian carcinomas, prostate cancer and non-small cell lung adenocarcinomas (2,4,30–32). Because increased levels of HA are observed in many cancer types, we conclude that an imbalance of HA synthesis and/or degradation may contribute to tumor progression. A previous study described siRNA-mediated knockdown of *HAS2* and the resulting reduction of cell growth and cellular migratory and invasive potentials in human breast cancer cells (8). Our data also showed that *HAS2* downregulation by 4-MU inhibited cell proliferation and motility in CF41.Mg cells. These data support the notion that repression of HA accumulation or overproduction is a useful target for therapy against breast cancer in humans and animals.

CD44, a major receptor for HA, is a transmembrane glycoprotein involved in cell-cell and cell-matrix interactions. HA-CD44 interactions activate cellular signaling pathways such as promotion of proliferation, survival, angiogenesis, migration and invasion of cancer cells (33,34). CD44 has also been identified as a marker of cancer stem cells in breast, head and neck, and colon cancer (35). CD44 has been implicated in human breast cancer tumor progression, although little is known about the pathological role of CD44 in canine mammary tumors. CD44 is preferentially expressed in benign canine mammary tumors and normal mammary tissue versus simple carcinomas and metastatic cells (36). However, our findings suggested that overproduction of HA induced proliferation, anti-apoptosis and cell motility. Therefore, our results support the notion that HA promotes tumor progression mediated by other receptors such as the receptor for HA-mediated motility (RHAMM), lymphatic vessel endothelial receptors (LYVE-1), and toll-like receptors 2 (TLR2) and 4 (TLR4) in canine mammary tumor.

EMT plays a key role during embryonic development, wound healing, tissue regeneration, organ fibrosis, and cancer metastasis (16). EMT is characterized by loss of cell-cell adhesion and cell polarity and increased migration of cancer cells. Other reports have demonstrated the correlation between drug resistance and EMT; it is now important to evaluate the effects of anticancer agents against mesenchymal-like cancer cells. The findings showed that 4-MU treatment of CF41.Mg cells, with the mesenchymal-like properties of canine mammary tumors, yielded marked growth retardation and apoptosis. Furthermore, 4-MU inhibited chemokinesis and chemotaxis of CF41.Mg cells. Chemokinesis plays a particularly important role in separation from the primary tumor mass, and the correlation between chemokinesis and EMT has been suggested (17). In addition, chemotaxis is associated with vascular invasion of cancer cells. The results suggest the possibility that 4-MU suppressed invasion and metastasis of canine mammary tumor cells.

It is well known that canine mammary tumors are more histologically complex than mammary tumors in humans. In addition, mesenchymal components such as myoepithelial cells are observed in many types of canine mammary tumors. Thus, it is often difficult to distinguish benign and malignant mammary tumors by cytological analyses in dogs (24). Our findings revealed that 4-MU effectively inhibited the growth and motility of CF41.Mg cells. A previous report also showed the anticancer effect of 4-MU on CF33 cells with epithelial properties. Therefore, the data suggest that 4-MU may be useful for wide-spectrum therapy of canine mammary tumors.

In summary, 4-MU blocked cell proliferation and cell migration mediated by downregulated *HAS2* mRNA expression in CF41.Mg cells. This study shows that 4-MU may be a potential agent for improved chemotherapy against breast cancers in dogs.
